# Multiple Nodes Co-Carrier Cooperative Transmission in LEO Communication Networks: Developing the Diversity Gain of Satellites

**DOI:** 10.3390/s24144533

**Published:** 2024-07-13

**Authors:** Tian Li, Guoyan Li, Xinwei Yue, Bin Dai

**Affiliations:** 1 The 54th Research Institute of China Electronics Technology Group Corporation, Shijiazhuang 050081, China; ljm0379@163.com; 2 School of Information and Communication Engineering, Beijing Information Science and Technology University, Beijing 100101, China; xinwei.yue@bistu.edu.cn; 3 School of Internet of Things, Nanjing University of Posts and Telecommunications, Nanjing 210003, China; daibin@njupt.edu.cn

**Keywords:** LEO communication networks, cooperative transmission, co-carrier transmission, multiple coverage

## Abstract

Low Earth orbit (LEO) satellite communication (SATCOM) networks have gradually been recognized as an efficient solution to enhance ground-based wireless networks. As one of the main characteristics of LEO SATCOM, the beam-edge area could be covered by multiple satellite nodes. In this case, user terminals (UTs) located at the beam-edge have the chance to connect one or more LEO satellites. To develop the diversity gain of multiple nodes in the overlapping area, we propose two high spectral efficiency cooperative transmission strategies, i.e., directly combining (DC) and selection combining (SC). In the DC scheme, signals arrived at the UT simultaneously could be combined into one enhanced signal. For downlink time division multiplexing, the SC scheme enables the UT to select the strongest signal path. Further, as there exists a significant channel gain difference of the beam-center and beam-edge areas, UTs in these two areas can be allocated in one resource block. In this case, we derive co-carriers based on DC and SC, respectively. To deeply analyze the novel methods, we study the ergodic sum-rate and outage probability while the outage diversity gain is further provided. Simulation results show that the co-carrier-based DC method has the ability to provide a higher ergodic sum-rate while the SC method performs better in terms of the outage probability.

## 1. Introduction

### 1.1. Literature Review

Due to its wide coverage characteristic, satellite communication (SATCOM) is able to provide long distance data transmission services. In 6G wireless communication systems, SATCOM has been recognized as an efficient way to spread terrestrial coverage [1,2]. Specifically, user terminals (UTs) can be expected to access the wireless network by connecting to the satellite. Satellites are able to relay the information directly to the target UT or to the gateway, which has wired connections with the ground-based networks [3]. To provide stable links between the satellite and UTs, SATCOM systems are often built on geostationary Earth orbit (GEO) satellites. Due to the extremely high signal path loss in GEO-based systems, large-size antennas are expected to be equipped at UTs. However, UTs are always designed in small-size terminals for Internet of Everything (IoE) uses. In this case, low Earth orbit (LEO) SATCOM has been widely studied in the scenario of IoE.

Since LEO satellites have the advantages of short transmission delay and low signal path loss, many companies have turned their attention to LEO SATCOM. As one of the pioneers, SpaceX launched the project of Starlink in 2014, which aims to built a SATCOM network with over ten thousand LEO satellites [4,5,6]. In the Starlink system, LEO satellites are connected by inter-satellite links. Then, multiple satellites could provide global service where users are able to connect to the internet by deploying a satellite terminal.

Dynamic strategies have been widely studied in SATCOM. The authors of [7] proposed a dynamic selection method in satellite-terrestrial networks where a power and server execution rate joint-optimization scheme is developed. UTs are able to access the proper node with the minimum expense. To alleviate the inter-beam interference in LEO SATCOM, ref. [8] proposed a dynamic beam illumination method. The number of simultaneously illuminated beams could be minimized while guaranteeing the coverage range.

One of the main concerns of the LEO SATCOM is determining how to efficiently develop the satellite resource, which has drawn significant attention. In this paper, we provide a literature review of high efficiency transmission methods under single and multiple nodes backgrounds.

High efficiency transmission methods have been widely discussed in existing works. In [9], the authors proposed a two time-slots transmission method in satellite-ground integrated systems. To fully utilize the channel diversity, the user with a strong channel condition could forward the useful information to the weak user after running the successive interference cancellation (SIC). In this case, the outage probability of the weak user could be improved. For the multi-beam scenario, the authors in [10] studied a non-orthogonal multiple access (NOMA)-based multi-beam transmission scheme in a terrestrial-satellite network. The beamforming vectors of the satellite and base stations were jointly optimized to boost the signal that arrived at the users. For the LEO NOMA, the authors in [11] proposed a channel condition feedback-based NOMA transmission method. To relieve the computational complexity onboard, LEO satellites generate random beams without calculating the exact beamforming vectors. Users could transmit the channel condition of each beam through the uplink channel. Then, the LEO could allocate users in the beams by considering the threshold of signal-to-interference-noise ratio (SINR). Generally speaking, the number of simultaneously generated beams on LEO satellites would be limited. In this case, our previous work in [12] studied a beamhopping-based NOMA transmission scheme where ground cells can be illuminated sequentially according to the requirements. We proposed a unified framework for code domain or power domain beamhopping NOMA. To satisfy the service requirements of users, the illuminating duration of beams and power factor are optimized. The work analyzed the outage behavior of beamhopping NOMA as well.

By cooperatively utilizing the advantages of GEO and LEO satellites, the SATCOM service could be significantly enhanced [13]. The GEO satellite is able to provide wide coverage while LEO satellites offer low latency service. In a GEO-LEO network, ground users have choices to access the GEO satellite or the LEO one. For the transmission techniques in GEO-LEO networks, the authors in [14] proposed an uplink NOMA in GEO-LEO network. In the novel scheme, ground users transmit signals to GEO and LEO satellites within one resource block. Simulation results showed that the ergodic sum-rate of NOMA is superior compared with the conventional orthogonal multiple access (OMA). Further, the ergodic sum-rate grows with the increase in elevation angle. To provide deep insight on the GEO-LEO transmission, our previous work in [15] proposed two uplink schemes for a two-user two-satellite scenario. The LEO satellite can serve beam-center and beam-edge users with one signal carrier by realizing the significant difference of the channel gain. As another solution, the beam-edge user can choose to access the GEO satellite while the beam-center user is still served by the LEO satellite. Simulation results found out that the LEO NOMA can provide a promising outage probability performance compared with the GEO-LEO OMA in low signal-to-noise ratio (SNR) regions. When the channel condition of the beam-center user is assumed to be superior to the beam-edge user, LEO NOMA outperforms the GEO-LEO OMA. In [16], the authors consider a downlink transmission method in a GEO and multiple LEO satellites scenario where ground users are classified as GEO users and LEO users. In detail, LEO users receive signals from LEO satellites by applying maximal ratio combining. To improve the LEO downlink transmission sum-rate, the signal power for each user is further optimized. Meanwhile, SINRs for GEO users are considered as optimization limitations in order to guarantee the quality of service.

Since the transmission environment of GEO SATCOM is significantly different from that of LEO SATCOM, a unified framework of GEO and LEO satellites is difficult to be realized—especially the physical layer. In this case, LEO-LEO cooperation has become a widely studied theme. The authors in [17] proposed a decoding protocol under multi-satellite background. At first, LEO satellites in a certain area could receive uplink signals simultaneously. Then, each satellite processes received signals and linearly maps to a value range. Further, all the processed signals would be transmitted to the gateway while the signals could be enhanced. Our previous work in [18] considered a three-user two-satellite scenario and developed a cooperative downlink NOMA scheme. Recognizing that there exists a huge channel gain gap between the beam-edge user and the beam-center user, the two satellites could serve two beam-center users and one beam-edge user cooperatively within the same resource block. The ergodic sum-rate showed the advancement of the new method. Under the same background, the authors in [19] investigated a cooperative NOMA in a Shadowed-Rician channel model. Outage probability is considered as the evaluation criteria where the superiority of NOMA is demonstrated by simulations. In addition to providing wide range communication, LEO constellation could also offer additional services like satellite positioning. The authors in [20] studied a multiple satellite cooperative positioning scheme where positioning accuracy for ground users could be enhanced by exploiting the diversity of the satellite. In detail, a UT receives useful signals from numerous satellites, and each satellite is able to generate several beams. Unfortunately, interference from other cells would be incurred since frequency reusing is applied onboard. To improve the positioning accuracy, the authors optimized the beamforming vectors and beam generating scheme. As one of the main concerns, carrying out full digital beamforming may not be possible since the computing ability onboard is quite limited.

Overall, the existing works mainly focused on the designing of low interference beamforming methods. UTs are assumed to be served by all satellites or specified satellites where selection is not involved. In addition, the advantages or disadvantages of accessing schemes have not yet been deeply analyzed.

### 1.2. Motivations and Contributions

Motivated by the working mechanism of LEO constellation, we study a multiple nodes cooperative transmission for downlink LEO SATCOM. Recognizing there exists a huge beam gain difference between the beam-edge UT and the beam-center UT, two co-carrier cooperative transmission methods are proposed where the outage probability and ergodic sum-rate are further analyzed.

The main contributions of the paper can be summarized as follows:

- Recognizing that UTs located at the beam-edge area could be covered by multiple satellites, we propose two promising cooperative transmission methods. In the first method, UTs receive multiple signal paths from numerous LEO nodes and combine them without processing selection. In this scheme, the receiver directly demodulates the received signals. To fully utilize the signals from different satellites, we then study a selection combination (SC) method where the strongest signal path is selected to perform the demodulation procedure.
- As there exists a significant beam gain difference at the beam-edge area and the beam-center area, we develop a co-carrier transmission-aided scheme based on the two cooperative methods. In detail, the beam-edge and beam-center UTs share the same resource block where the signals for these two users would be superposed as one signal after modulation. To separate the two signals, SIC is applied at the receiver.
- In order to evaluate the performance of the new methods, we derive the expressions of the ergodic sum-rate and outage probability. Moreover, we further consider the processing expense of the SC algorithm. At last, simulation results confirm the performance of the work.
- Both the proposed methods are energy efficient. The received signal of the co-carrier-based direct combination (DC) method could be enhanced by combining signals from multiple satellites without increasing the transmit power, while the co-carrier-based SC scheme enables the UT access to the satellite with the best channel condition.

The rest of the paper is organized as follows. Section 2 describes the system model of a cooperative LEO SATCOM. In Section 3, the DC and SC methods are proposed while the co-carrier transmission is studied as well. To evaluate the performance, expressions of ergodic sum-rate and outage probability are derived and analyzed theoretically in Section 4. The impact of processing expense of the SC method is also studied. Simulation results of the ergodic sum-rate and outage probability are presented and discussed in Section 5, and the paper is concluded in Section 6.

*Notation*: Vectors and matrices are presented by lowercase and uppercase bold letters, respectively. The transpose and Hermitian transpose are denoted by superscripts T and H, respectively. The statistical expectation and the probability are presented by E[·] and P(·), respectively. While CN(a,b) denotes the distribution of circularly symmetric complex Gaussian (CSCG) random variables with mean *a* and covariance *b*.

## 2. System Model

**Figure 1:**
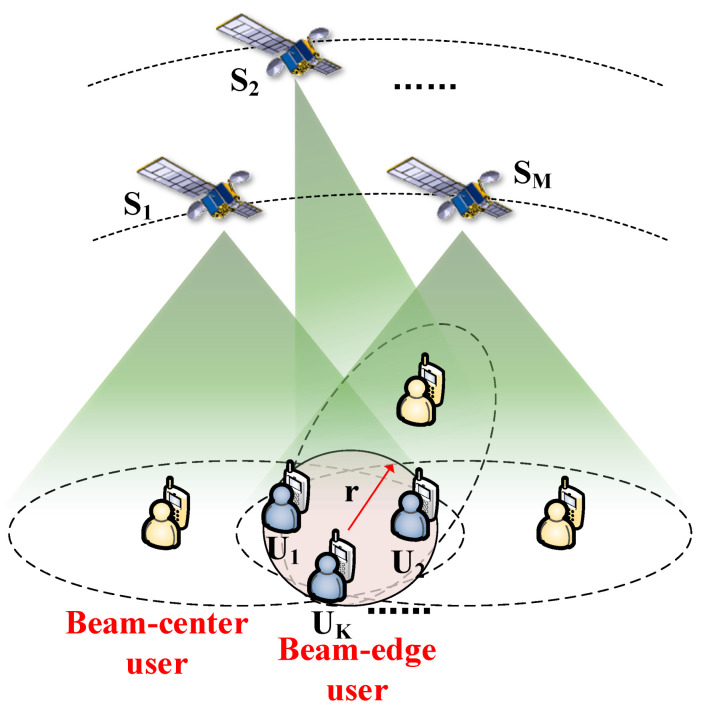
System model.

In this paper, we consider a multiple LEO satellites multiple UTs communication scenario, shown in Figure 1. The symbols used in the system are mainly summarized in Table 1.

Since there exists large numbers of satellites in an LEO constellation system, the beam-edge area can be covered by several LEO nodes. The radius of the overlapping area, covered by *M* satellites, is assumed to be *r*. Specifically, *K* UTs are allocated in the area. To flexibly adjust the beam direction, yz-plane uniform planar phased array (UPA) with half-wavelength spacing is applied on the satellite. The number of array elements is assumed to be $N_{y}^{S}=N_{z}^{S}$. Let ϕ and θ denote the azimuth and elevation angles of departure at the satellite and λ is the wavelength. Then, the array response vector at the satellite can be derived as [21]

(1) $$\begin{array}{c} a\left(\phi ,\theta \right)=\frac{1}{\sqrt{N_{y}^{S}N_{z}^{S}}}{\left[1,\ldots ,e^{jt\mu (m\sin(\phi )\sin(\theta )+n\cos(\theta ))},\ldots ,e^{jt\mu (\left(N_{y}^{S}-1\right)\sin\left(\phi \right)\sin\left(\theta \right)+\left(N_{z}^{S}-1\right)\cos\left(\theta \right))}\right]}^{T}, \end{array}$$

where t=2π/λ and μ=λ/2.

Since the space-ground links contain large-scale and small-scale fading in LEO SATCOM, the downlink channel from satellite *m* to UT *k* can be modeled as

(2) $$\begin{array}{c} h_{k,m}^{H}=\rho_{k,m}l\left(d_{k,m}\right)\sqrt{E_{S}}a^{H}\left(\phi_{k,m},\theta_{k,m}\right), \end{array}$$

where $l\left(d_{k,m}\right)=\sqrt{{\left(\lambda /\left(4\pi d_{k,m}\right)\right)}^{2}}$, $d_{k,m}$ represents the distance between the satellite and UT, $\rho_{k,m}∼\mathrm{CN}\left(0,1\right)$ denotes the small-scale fading channel coefficient, $\phi_{k,m}$ and $\theta_{k,m}$ denote the azimuth and elevation angles of departure from satellite *m* to UT *k*, respectively. In detail, $E_{S}$ is the normal gain of transmit beams.

## 3. Multiple Nodes Cooperative Transmission

### 3.1. Co-Carrier-Based Direct Combination

**Figure 2:**
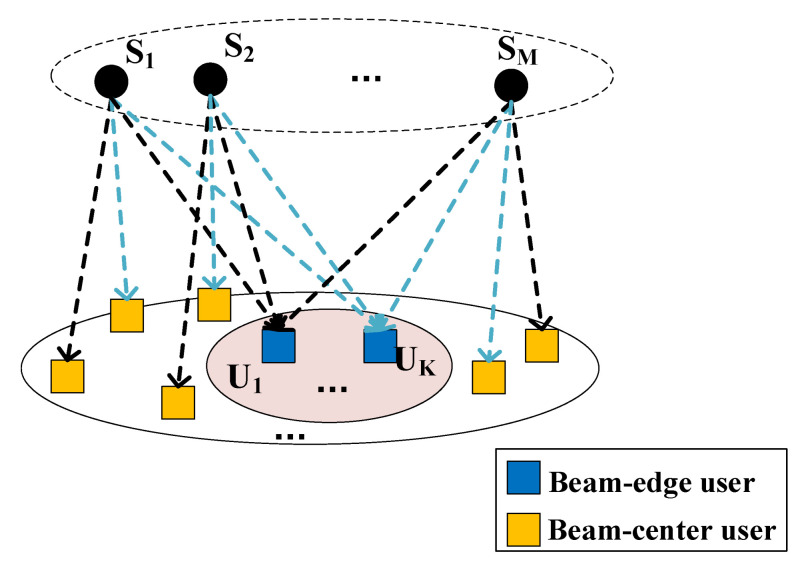
Co-carrier transmission in LEO constellation.

Since the equivalent channel gain of the beam-edge user and the beam-center user has a significant difference in LEO SATCOM [15], we propose a co-carrier transmission method shown in Figure 2.

UTs in the overlapping area can receive multiple signal paths from the *M* satellites. To enhance the signal strength, we propose a co-carrier-based DC scheme where UTs receive *M* path signals and directly superpose them. In the co-carrier-based scheme, one pair of beam-edge and beam-center UTs is allocated with one signal carrier at one time-slot. Multiple UT pairs could be served by allocating more carrier and time-slot resources.

For UT *k* in the overlapping area, the received signal can be expressed as

(3) $$\begin{array}{c} y_{k}=\sum_{m=1}^{M}h_{k,m}^{H}w_{m}\left(s_{k}+x_{m}\right)+n, \end{array}$$

where $w_{m}$ is the beamforming vector of the *m*-th satellite. $s_{k}$ represents the signal for UT *k* in the overlapping area, $x_{m}$ denotes the signal for the beam-center UT under the *m*-th satellite. The background noise with power spectral density $\sigma^{2}$ is denoted as $n∼\mathrm{CN}(0,\sigma^{2})$. Note that the effect of Doppler frequency shift could be relieved by applying the satellite ephemeris-based frequency offset pre-compensation scheme.

Assume $E[|s_{k}{|}^{2}]=P_{k}$ and $E[|x_{m}{|}^{2}]=P_{m}$. To guarantee the fairness for the beam-edge and beam-center UTs, we have $P_{m}=\alpha P_{k}$, where 0<α<1. Since $s_{k}$ is stronger compared with $x_{m}$, the useful information could be directly demodulated at UT *k* where the SINR can be derived as

(4) $$\begin{array}{c} SINR_{k,k}=\frac{|\sum_{m=1}^{M}h_{k,m}^{H}w_{m}{|}^{2}P_{k}}{\sum_{m=1}^{M}{\left|h_{k,m}^{H}w_{m}\right|}^{2}P_{m}+\sigma^{2}}. \end{array}$$

We apply SIC at UT *m*, the SINR of $s_{k}$ can be calculated as

(5) $$\begin{array}{c} SINR_{m,k}=\frac{|h_{m,m}^{H}w_{m}{|}^{2}P_{k}}{|h_{m,m}^{H}w_{m}{|}^{2}P_{m}+\sigma^{2}}. \end{array}$$

After processing SIC, the SINR of $x_{m}$ is

(6) $$\begin{array}{c} SNR_{m,m}=\frac{|h_{m,m}^{H}w_{m}{|}^{2}P_{m}}{\sigma^{2}}. \end{array}$$

Thus, the SINR of UT *k* satisfies $SINR_{k}=\min\left\{SINR_{k,k},SINR_{1,k},\ldots ,SINR_{m,k},SINR_{M,k}\right\}\;=SINR_{k,k}$.

### 3.2. Co-Carrier-Based Selection Combination

To fully utilize the multiple signals from the *M* satellites, we propose SC with co-carrier transmission method in this section. In SC, UTs are able to receive *M* superposed signals in *M* time slots, and select one path with the best channel condition. Under this mechanism, signals at the *k*-th UT can be expressed as

(7) $$\begin{array}{c} y_{k,1}^{{\;}^{\prime }}=h_{k,1}^{H}w_{1}\left(s_{k}+x_{1}\right)+n,\\ \ldots ,\\ y_{k,M}^{{\;}^{\prime }}=h_{k,M}^{H}w_{M}\left(s_{k}+x_{M}\right)+n, \end{array}$$

where $y_{k,m}^{{\;}^{\prime }}$ denotes the signal of the *m*-th satellite arrived at UT-*k*.

According to the co-carrier transmission scheme, SINRs at UT *k* can be calculated as

(8) $$\begin{array}{c} SINR_{1,k}^{{\;}^{\prime }}=\frac{|h_{k,1}^{H}w_{1}{|}^{2}P_{k}}{|h_{k,1}^{H}w_{1}{|}^{2}P_{1}+\sigma^{2}},\\ \ldots ,\\ SINR_{M,k}^{{\;}^{\prime }}=\frac{|h_{k,M}^{H}w_{M}{|}^{2}P_{k}}{|h_{k,M}^{H}w_{M}{|}^{2}P_{M}+\sigma^{2}}. \end{array}$$

To obtain a better system performance, we select the strongest signal path for transmission. In this case, we have $SINR_{k}^{{\;}^{\prime }}=\max\left\{SINR_{1,k}^{{\;}^{\prime }},\ldots ,SINR_{M,k}^{{\;}^{\prime }}\right\}$. Assume the *m*-th satellite provides the best SINR. After SIC, SNR of the beam-center UT under the *m*-th satellite is the same with (6). In this paper, we assume $P_{1}=\ldots =P_{M}=P$.

## 4. Performance Analysis

Considering the processing capability onboard is weak, we apply the beam steering method for signal transmission. For convenience reasons, we provide the following assumption.

**Assumption 1.**  *For the beam steering method, the equivalent downlink channel gain can be assumed as*

(9) $$\begin{array}{cc} g_{k,m} & =h_{k,m}^{H}w_{m}\\ \; & =\rho_{k,m}l\left(d_{k,m}\right)\sqrt{E_{S}}a^{H}\left(\phi_{k,m},\theta_{k,m}\right)\times a\left(\phi_{k,m},\theta_{k,m}\right)/||a\left(\phi_{k,m},\theta_{k,m}\right){\left|\right|}^{2}\\ \; & =\rho_{k,m}l\left(d_{k,m}\right)\sqrt{E_{S}\left(\eta_{k,m}\right)}, \end{array}$$

*where $\eta_{k,m}$ denotes the angle between UT k and beam-center compared to satellite m. In detail, $E_{S}\left(\eta_{k,m}\right)=E_{S}{\left(\frac{J_{1}\left(u_{k,m}\right)}{2u_{k,m}}+36\frac{J_{3}\left(u_{k,m}\right)}{u_{k,m}^{3}}\right)}^{2}$ [19]. Here, $u_{k,m}=2.07123\frac{\sin(\eta_{k,m})}{\sin(\eta_{3dB})}$, $\eta_{3dB}$ denotes the angle of 3 dB for the transmit beam.*

### 4.1. Ergodic Rate

#### 4.1.1. Co-Carrier-Based Direct Combination

Under Assumption 1, we have $|g_{k,m}\left|=\right|\rho_{k,m}||l\left(d_{k,m}\right)\sqrt{E_{S}\left(\eta_{k,m}\right)}\left|=\right|\rho_{k,m}\left|\right|\beta_{k,m}|$, where $g_{k,m}∼\mathrm{CN}(0,|\beta_{k,m}{|}^{2})$. Similarly, we can derive $g_{m,m}∼\mathrm{CN}(0,|\beta_{m,m}{|}^{2})$ for the beam-center user under satellite *m*. In this case, SINR of the *k*-th UT in (4) can be further computed as

(10) $$\begin{array}{c} SINR_{k}=\frac{|\sum_{m=1}^{M}g_{k,m}{|}^{2}P_{k}}{\sum_{m=1}^{M}{\left|g_{k,m}\right|}^{2}P+\sigma^{2}}. \end{array}$$

Further, the SNR of the beam-center UT can be calculated as

(11) $$\begin{array}{c} SNR_{m,m}=\frac{|g_{m,m}{|}^{2}P}{\sigma^{2}}. \end{array}$$

Then, the achievable sum-rate of UT *k* can be expressed as

(12) $$\begin{array}{c} R_{k}=\log_{2}\left(1+SINR_{k}\right)=\log_{2}\left(1+\frac{|\sum_{m=1}^{M}g_{k,m}{|}^{2}P_{k}}{\sum_{m=1}^{M}{\left|g_{k,m}\right|}^{2}P+\sigma^{2}}\right). \end{array}$$

Accordingly, we have

(13) $$\begin{array}{c} R_{m}=\log_{2}\left(1+SINR_{m,m}\right)=\log_{2}\left(1+\frac{|g_{m,m}{|}^{2}P}{\sigma^{2}}\right). \end{array}$$

Since UT *k* could receive *M* signal paths from the *M* satellites, and each satellite could serve one beam-center UT simultaneously by applying the co-carrier-based DC method with one resource block. The ergodic sum-rate of the co-carrier-based DC method can be expressed as $E\left[R_{DC}\right]=E\left[R_{k}\right]+\sum_{m=1}^{M}E\left[R_{m}\right]$. Let $\delta =P_{k}/\sigma^{2}$, $|\beta_{k,1}{|}^{2}=\ldots =|\beta_{k,M}{|}^{2}={\left|\beta_{k}\right|}^{2}$, and $|\beta_{1,1}{|}^{2}=\ldots =|\beta_{M,M}{|}^{2}={\left|\beta_{m}\right|}^{2}$. Then, we can derive the following theorem.

**Theorem 1.**  *The ergodic sum-rate of UT k and the co-carrier-based DC method in multiple nodes LEO SATCOM can be given as*

(14) $$\begin{array}{cc} E[R_{DC}] & =E\left[R_{k}\right]+\sum_{m=1}^{M}E\left[R_{m}\right]\\ \; & =\frac{\delta M|\beta_{k}{|}^{2-2M}}{\ln2}\sum_{i=1}^{n}\omega_{i}B_{k}\left(\delta M\right|\beta_{k}{|}^{2}x_{i})-\frac{Me^{1/(|\beta_{m}{|}^{2}\alpha \delta )}}{\ln2}\mathrm{Ei}\left(-\frac{1}{|\beta_{m}{|}^{2}\alpha \delta }\right), \end{array}$$

*where $B_{k}\left(x\right)=\frac{\left(x\alpha /(M\right|\beta_{k}{|}^{2}{)+\frac{1}{|\beta_{k}{|}^{2}})}^{-M}}{1+x}$, $x_{i}$ and $\omega_{i}$ denote the Gauss-Laguerre quadrature nodes and weights over [0,+∞] [22]. To be specific, $\mathrm{Ei}\left(x\right)=-\int_{-x}^{\infty }\frac{e^{-r}}{r}dr$ is the exponential integral function [23].*

**Proof.**  For notational convenience, we rewrite the ergodic rate for UT *k* as $R_{k}=\log_{2}\left(1+\frac{\overset{Y}{\overset{︷}{|\sum_{m=1}^{M}g_{k,m}{|}^{2}}}P_{k}}{\overset{Z}{\overset{︷}{\sum_{m=1}^{M}{\left|g_{k,m}\right|}^{2}}}P+\sigma^{2}}\right)$. Specifically, $\sum_{m=1}^{M}g_{k,m}∼\mathrm{CN}(0,M|\beta_{k}{|}^{2})$. In this case, we have $Y=|\sum_{m=1}^{M}g_{k,m}{|}^{2}∼\exp(1/(M|\beta_{k}{|}^{2}))$ whose probability density function (PDF) is

(15) $$\begin{array}{c} f_{Y}\left(y\right)=\frac{1}{M|\beta_{k}{|}^{2}}e^{-\frac{y}{M|\beta_{k}{|}^{2}}}. \end{array}$$

Since $g_{k,m}$s are independent and identically distributed, the PDF of *Z* can be calculated as

(16) $$\begin{array}{c} f_{Z}\left(z\right)=\frac{\left(z^{M-1}e^{-1/|\beta_{k}{|}^{2}z}\right)\left(1/\right|\beta_{k}{{|}^{2})}^{M}}{(M-1)!}. \end{array}$$

Then, $E[R_{k}]$ is given by

(17) $$\begin{array}{c} E\left[R_{k}\right]=E\left[\log_{2}\left(1+\frac{YP_{k}}{ZP+\sigma^{2}}\right)\right]=\frac{1}{\ln2}\int_{0}^{\infty }\frac{1-F_{X}\left(x\right)}{1+x}dx, \end{array}$$

where $X=\frac{YP_{k}}{ZP+\sigma^{2}}$ and $F_{X}\left(x\right)$ is the cumulative distribution function (CDF). According to the PDF of *Y* and *Z*, $F_{X}\left(x\right)$ can be further computed as

(18) $$\begin{array}{cc} F_{X}\left(x\right) & =\mathrm{Pr}(\frac{YP_{k}}{ZP+\sigma^{2}}<x)\\ \; & =\mathrm{Pr}(Y\leq \frac{x(ZP+\sigma^{2})}{P_{k}})\\ \; & =\int_{0}^{\infty }\int_{0}^{\frac{x(ZP+\sigma^{2})}{P_{k}}}f_{Y}\left(y\right)dyf_{Z}\left(z\right)dz\\ \; & =\int_{0}^{\infty }\left(1-e^{-\frac{x(ZP+\sigma^{2})}{M|\beta_{k}{|}^{2}P_{k}}}\right)f_{Z}\left(z\right)dz\\ \; & =1-\int_{0}^{\infty }e^{-\frac{x(ZP+\sigma^{2})}{M|\beta_{k}{|}^{2}P_{k}}}f_{Z}\left(z\right)dz\\ \; & =1-\frac{\left(1/\right|\beta_{k}{{|}^{2})}^{M}}{(M-1)!}\int_{0}^{\infty }z^{M-1}e^{-\frac{x(ZP+\sigma^{2})}{M|\beta_{k}{|}^{2}P_{k}}-\frac{z}{|\beta_{k}{|}^{2}}}dz. \end{array}$$

According to ([23], 3.351.3), (18) can be further calculated as

(19) $$\begin{array}{c} F_{X}\left(x\right)=1-(1/|\beta_{k}{{|}^{2})}^{M}{\left(\frac{xP}{M|\beta_{k}{|}^{2}P_{k}}+\frac{1}{|\beta_{k}{|}^{2}}\right)}^{-M}e^{-\frac{x\sigma^{2}}{M|\beta_{k}{|}^{2}P_{k}}}. \end{array}$$

Then, (17) can be derived as

(20) $$\begin{array}{c} E\left[R_{k}\right]=\frac{\left(1/\right|\beta_{k}{{|}^{2})}^{M}}{\ln2}\int_{0}^{\infty }\frac{{\left(\frac{x\alpha }{M|\beta_{k}{|}^{2}}+\frac{1}{|\beta_{k}{|}^{2}}\right)}^{-M}e^{-\frac{x}{M|\beta_{k}{|}^{2}\delta }}}{1+x}dx. \end{array}$$

Let $B_{k}\left(x\right)=\frac{{\left(\frac{x\alpha }{M|\beta_{k}{|}^{2}}+\frac{1}{|\beta_{k}{|}^{2}}\right)}^{-M}}{1+x}$. Therefore, (20) becomes

(21) $$\begin{array}{cc} E[R_{k}] & =\frac{\left(1/\right|\beta_{k}{{|}^{2})}^{M}}{\ln2}\int_{0}^{\infty }e^{-\frac{x}{\delta M|\beta_{k}{|}^{2}}}B_{k}\left(x\right)dx\\ \; & \overset{\left(a\right)}{=}\frac{\delta M|\beta_{k}{|}^{2-2M}}{\ln2}\sum_{i=1}^{n}\omega_{i}B_{k}\left(\delta M\right|\beta_{k}{|}^{2}x_{i}), \end{array}$$

where (a) follows Gauss-Laguerre quadrature, and $x_{i}$ and $\omega_{i}$ denote the Gauss-Laguerre quadrature nodes and weights over [0,+∞].

Next, we move on to the ergodic rate of the beam-center user, where $E[R_{m}]$ can be calculated as

(22) $$\begin{array}{c} E\left[R_{m}\right]=E\left[\log_{2}\left(1+\frac{|g_{m,m}{|}^{2}P}{\sigma^{2}}\right)\right]=\frac{1}{\ln2}\int_{0}^{\infty }\frac{1-F_{X}\left(x\right)}{1+x}dx, \end{array}$$

where $X=\frac{|g_{m,m}{|}^{2}P}{\sigma^{2}}$. Since $|g_{m,m}{|}^{2}∼\exp(1/|\beta_{m}{|}^{2})$, we have

(23) $$\begin{array}{cc} F_{X}\left(x\right) & =\mathrm{Pr}(\frac{|g_{m,m}{|}^{2}P}{\sigma^{2}}\leq x)\\ \; & =\mathrm{Pr}(|g_{m,m}{|}^{2}\leq \frac{x\sigma^{2}}{P})\\ \; & =1-e^{-\frac{x}{\alpha \delta |\beta_{m}{|}^{2}}}. \end{array}$$

Substituting (23) into (22), we can derive

(24) $$\begin{array}{cc} E[R_{m}] & =\frac{1}{\ln2}\int_{0}^{\infty }\frac{1-(1-e^{-\frac{x}{\alpha \delta |\beta_{m}{|}^{2}}})}{1+x}dx\\ \; & =\frac{1}{\ln2}\int_{0}^{\infty }\frac{e^{-\frac{x}{\alpha \delta |\beta_{m}{|}^{2}}}}{1+x}dx\\ \; & \overset{\left(b\right)}{=}-\frac{1}{\ln2}e^{\frac{1}{\alpha \delta |\beta_{m}{|}^{2}}}\mathrm{Ei}\left(-\frac{1}{\alpha \delta |\beta_{m}{|}^{2}}\right), \end{array}$$

where (b) follows ([23], 3.352.4). From (24) and (21), we can obtain (14).

The theorem is proved. □

#### 4.1.2. Co-Carrier-Based Selection Combination

**Figure 3:**
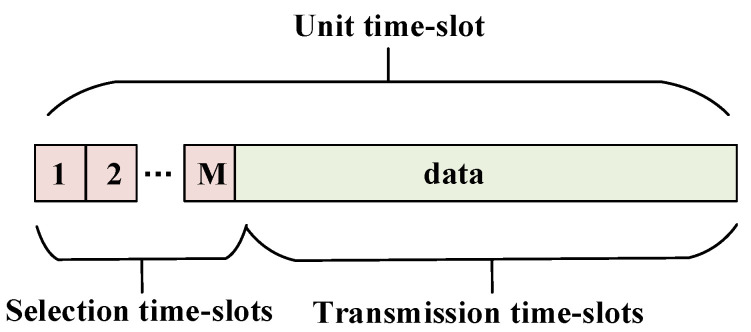
Co-carrier-based SC method.

In the co-carrier-based SC method, shown in Figure 3, UT *k* would select the signal path with the highest SINR and the corresponding beam-center UT could be paired.

Assume that the selection time-slot per signal is τ. Then, the ergodic sum-rate of the SC method can be expressed as $E\left[R_{SC}\right]=E\left[R_{k}^{{\;}^{\prime }}\right]+E\left[R_{m}^{{\;}^{\prime }}\right]$, where $E\left[R_{k}^{{\;}^{\prime }}\right]=\left(1-M\tau \right)\log_{2}\left(1+\max\left\{SINR_{1,k}^{{\;}^{\prime }},\ldots ,SINR_{M,k}^{{\;}^{\prime }}\right\}\right)$ and $E\left[R_{m}^{{\;}^{\prime }}\right]=\left(1-M\tau \right)E\left[R_{m}\right]$. The ergodic sum-rate can be further derived in the following theorem.

**Theorem 2.**  *The ergodic sum-rate of UT k of the co-carrier-based SC method in multiple nodes LEO SATCOM can be given as*

(25) $$\begin{array}{c} E\left[R_{SC}\right]=\left(1-M\tau \right)\left(\frac{1}{\ln2}\int_{0}^{\infty }\frac{1-{\left(1-e^{-\frac{x}{|\beta_{k}{|}^{2}\delta \left(1-\alpha x\right)}}\right)}^{M}}{1+x}dx-\frac{1}{\ln2}e^{\frac{1}{\alpha \delta |\beta_{m}{|}^{2}}}\mathrm{Ei}\left(-\frac{1}{\alpha \delta |\beta_{m}{|}^{2}}\right)\right). \end{array}$$

**Proof.**  Let $X=\max\{SINR_{1,k}^{{\;}^{\prime }},\ldots ,SINR_{M,k}^{{\;}^{\prime }}\}$. Then, the CDF of *X* can be calculated as

(26) $$\begin{array}{cc} F_{X}\left(x\right) & =\mathrm{Pr}(\max\left\{SINR_{1,k}^{{\;}^{\prime }},\ldots ,SINR_{M,k}^{{\;}^{\prime }}\right\}\leq x)\\ \; & =\mathrm{Pr}(SINR_{1,k}^{{\;}^{\prime }}\leq x,\ldots ,SINR_{M,k}^{{\;}^{\prime }}\leq x)\\ \; & =\mathrm{Pr}(\frac{|g_{k,1}{|}^{2}\delta }{|g_{k,1}{|}^{2}\alpha \delta +1}\leq x,\cdots ,\frac{|g_{k,M}{|}^{2}\delta }{|g_{k,M}{|}^{2}\alpha \delta +1}\leq x)\\ \; & =\left(1-e^{-\frac{x}{|\beta_{k,1}{|}^{2}\delta \left(1-\alpha x\right)}}\right),\ldots ,\left(1-e^{-\frac{x}{|\beta_{k,M}{|}^{2}\delta \left(1-\alpha x\right)}}\right)\\ \; & ={\left(1-e^{-\frac{x}{|\beta_{k}{|}^{2}\delta \left(1-\alpha x\right)}}\right)}^{M}. \end{array}$$

Consequently, we have

(27) $$\begin{array}{c} E\left[R_{k}^{{\;}^{\prime }}\right]=\frac{1-M\tau }{\ln2}\int_{0}^{\infty }\frac{1-F_{X}\left(x\right)}{1+x}dx=\frac{1-M\tau }{\ln2}\int_{0}^{\infty }\frac{1-{\left(1-e^{-\frac{x}{|\beta_{k}{|}^{2}\delta \left(1-\alpha x\right)}}\right)}^{M}}{1+x}dx. \end{array}$$

Similarly, $E[R_{m}]$ is the same as (24), and we can easily obtain $E[R_{m}^{{\;}^{\prime }}]$.

The theorem is proved. □

### 4.2. Outage Probability

As another assessing metric, we also analyze how the proposed methods behave in terms of the outage probability. In this paper, we mainly focus on the outage probability of UTs located in the overlapping area.

#### 4.2.1. Co-Carrier-Based Direct Combination

Let $\Gamma_{k}$ denote the demodulation threshold of UT *k*. The outage probability of UT *k* for the co-carrier-based DC is given below.

**Theorem 3.**  *The outage probability of the k-th UT in the co-carrier-based DC scheme is given by*

(28) $$\begin{array}{c} {\mathrm{Pr}}_{k}=1-(1/|\beta_{k}{{|}^{2})}^{M}{\left(\frac{\alpha \Gamma_{k}}{M|\beta_{k}{|}^{2}}+\frac{1}{|\beta_{k}{|}^{2}}\right)}^{-M}e^{-\frac{\Gamma_{k}}{\delta M|\beta_{k}{|}^{2}}}. \end{array}$$

**Proof.**  The outage probability of UT *k* can be defined as ${\mathrm{Pr}}_{k}=P\left(SINR_{k}<\Gamma_{k}\right)$, which can be further calculated as

(29) $$\begin{array}{cc} {\mathrm{Pr}}_{k} & =P(\frac{|\sum_{m=1}^{M}g_{k,m}{|}^{2}P_{k}}{\sum_{m=1}^{M}{\left|g_{k,m}\right|}^{2}P+\sigma^{2}}<\Gamma_{k})\\ \; & =P(\frac{YP_{k}}{ZP+\sigma^{2}}<\Gamma_{k})\\ \; & =F_{X}\left(\Gamma_{k}\right), \end{array}$$

where $X=\frac{YP_{k}}{ZP+\sigma^{2}}$.

Following (19), (29) can be derived as

(30) $$\begin{array}{c} F_{X}\left(\Gamma_{k}\right)=1-(1/|\beta_{k}{{|}^{2})}^{M}{\left(\frac{\alpha \Gamma_{k}}{M|\beta_{k}{|}^{2}}+\frac{1}{|\beta_{k}{|}^{2}}\right)}^{-M}e^{-\frac{\Gamma_{k}}{\delta M|\beta_{k}{|}^{2}}}. \end{array}$$

The theorem is proved. □

Next, we analyze the outage probability of the beam-center UT. Assume that the demodulation threshold is $\Gamma_{m}$. The outage occurs when $SINR_{m,k}<\Gamma_{k}$ or $SNR_{m,m}<\Gamma_{m}$ since the beam-center UT should decode $s_{k}$ and $x_{m}$ successively. Then, we derive the following theorem.

**Theorem 4.**  *The outage probability of the beam-center UT in the co-carrier-based DC scheme is given by*

(31) $$\begin{array}{c} {\mathrm{Pr}}_{m}=\left\{\begin{array}{cc} 1-e^{-\frac{\Gamma_{m}}{\alpha \delta |\beta_{m}{|}^{2}}}, & \frac{\Gamma_{k}}{1-\alpha \Gamma_{k}}\leq \frac{\Gamma_{m}}{\alpha }\\ 1-e^{-\frac{\Gamma_{k}}{\delta |\beta_{m}{|}^{2}\left(1-\alpha \Gamma_{k}\right)}}, & \frac{\Gamma_{k}}{1-\alpha \Gamma_{k}}>\frac{\Gamma_{m}}{\alpha } \end{array}\right. \end{array}$$

**Proof.**  The outage probability of the beam-center UT can be expressed as

(32) $$\begin{array}{cc} {\mathrm{Pr}}_{m} & =P(SINR_{m,k}<\Gamma_{k}\;or\;SNR_{m,m}<\Gamma_{m})\\ \; & =1-P(\frac{|g_{m,m}{|}^{2}P_{k}}{|g_{m,m}{|}^{2}P_{m}+\sigma^{2}}\geq \Gamma_{k},\frac{|g_{m,m}{|}^{2}P_{m}}{\sigma^{2}}\geq \Gamma_{m})\\ \; & =1-P(|g_{m,m}{|}^{2}\geq \max\left\{\frac{\Gamma_{k}}{\delta -\Gamma_{k}\alpha \delta },\frac{\Gamma_{m}}{\alpha \delta }\right\}), \end{array}$$

which can be further discussed as

(33) $$\begin{array}{cc} {\mathrm{Pr}}_{m} & =1-P(|g_{m,m}{|}^{2}\geq \max\left\{\frac{\Gamma_{k}}{\delta -\Gamma_{k}\alpha \delta },\frac{\Gamma_{m}}{\alpha \delta }\right\})\\ \; & =\left\{\begin{array}{cc} 1-P(|g_{m,m}{|}^{2}\geq \frac{\Gamma_{m}}{\alpha \delta }), & \frac{\Gamma_{k}}{1-\alpha \Gamma_{k}}\leq \frac{\Gamma_{m}}{\alpha }\\ 1-P(|g_{m,m}{|}^{2}\geq \frac{\Gamma_{k}}{\delta -\Gamma_{k}\alpha \delta }), & \frac{\Gamma_{k}}{1-\alpha \Gamma_{k}}>\frac{\Gamma_{m}}{\alpha } \end{array}\right. \end{array}$$

When $\frac{\Gamma_{k}}{1-\alpha \Gamma_{k}}\leq \frac{\Gamma_{m}}{\alpha }$, we have

(34) $$\begin{array}{c} P(|g_{m,m}{|}^{2}\geq \frac{\Gamma_{m}}{\alpha \delta })=1-P(|g_{m,m}{|}^{2}<\frac{\Gamma_{m}}{\alpha \delta })=e^{-\frac{\Gamma_{m}}{\alpha \delta |\beta_{m}{|}^{2}}}. \end{array}$$

Similarly, we can derive

(35) $$\begin{array}{c} P(|g_{m,m}{|}^{2}\geq \frac{\Gamma_{k}}{\delta -\Gamma_{k}\alpha \delta })=1-P(|g_{m,m}{|}^{2}<\frac{\Gamma_{k}}{\delta -\Gamma_{k}\alpha \delta })=e^{-\frac{\Gamma_{k}}{\delta |\beta_{m}{|}^{2}\left(1-\alpha \Gamma_{k}\right)}}. \end{array}$$

for $\frac{\Gamma_{k}}{1-\alpha \Gamma_{k}}>\frac{\Gamma_{m}}{\alpha }$.

The theorem is proved. □

#### 4.2.2. Co-Carrier-Based Selection Combination

For the outage probability of UT *k* in the co-carrier-based SC scheme, we have the following theorem.

**Theorem 5.**  *The outage probability of the k-th beam-edge UT in the co-carrier-based SC scheme is given by*

(36) $$\begin{array}{c} {\mathrm{Pr}}_{k}^{{\;}^{\prime }}=\left\{\begin{array}{cc} {\left(1-e^{-\frac{\Gamma_{k}}{|\beta_{k}{|}^{2}\delta \left(1-\alpha \Gamma_{k}\right)}}\right)}^{M}, & 1-\Gamma_{k}\alpha >0\\ 1, & 1-\Gamma_{k}\alpha \leq 0 \end{array}\right. \end{array}$$

**Proof.**  The outage probability of UT *k* in SC method can be defined as

(37) $$\begin{array}{cc} {\mathrm{Pr}}_{k}^{{\;}^{\prime }} & =P(SINR_{k}^{{\;}^{\prime }}<\Gamma_{k})\\ \; & =P(\max\left\{SINR_{1,k}^{{\;}^{\prime }},\ldots ,SINR_{M,k}^{{\;}^{\prime }}\right\}<\Gamma_{k})\\ \; & =P\left(\frac{|g_{k,1}{|}^{2}\delta }{|g_{k,1}{|}^{2}\alpha \delta +1}<\Gamma_{k}\right),\cdots ,P\left(\frac{|g_{k,M}{|}^{2}\delta }{|g_{k,M}{|}^{2}\alpha \delta +1}<\Gamma_{k}\right). \end{array}$$

According to (26), we can derive (36).

The theorem is proved. □

Since the beam-center UT in the co-carrier-based SC method receives one signal path as well, the outage probability of beam-center UT is the same as that in the co-carrier-based DC method shown in (31).

#### 4.2.3. Diversity Order Analysis

To deeply study the outage performance of the two methods, we analyze the diversity order which is defined as $D=-\lim_{\delta \to \infty }\frac{\log({\mathrm{Pr}}^{\infty }\left(\delta \right))}{\log\delta }$.

As $e^{-x}\approx 1-x$ when x→0, (28) of the co-carrier-based DC method can be further derived as

(38) $$\begin{array}{cc} \; & {\mathrm{Pr}}_{k}=1-(1/|\beta_{k}{{|}^{2})}^{M}{\left(\frac{\alpha \Gamma_{k}}{M|\beta_{k}{|}^{2}}+\frac{1}{|\beta_{k}{|}^{2}}\right)}^{-M}e^{-\frac{\Gamma_{k}}{\delta M|\beta_{k}{|}^{2}}}\\ \; & \overset{\delta \to \infty }{\approx }1-(1/|\beta_{k}{{|}^{2})}^{M}{\left(\frac{\alpha \Gamma_{k}}{M|\beta_{k}{|}^{2}}+\frac{1}{|\beta_{k}{|}^{2}}\right)}^{-M}\left(1-\frac{\Gamma_{k}}{\delta M|\beta_{k}{|}^{2}}\right). \end{array}$$

In this case, the diversity order can be found as D=0.

For the co-carrier-based selection combination, ${\left(1-e^{-\frac{\Gamma_{k}}{|\beta_{k}{|}^{2}\delta \left(1-\alpha \Gamma_{k}\right)}}\right)}^{M}\overset{\delta \to \infty }{\approx }{\left(\frac{\Gamma_{k}}{|\beta_{k}{|}^{2}\delta \left(1-\alpha \Gamma_{k}\right)}\right)}^{M}$. Then, $D^{{\;}^{\prime }}=M$.

Consequently, we can clearly find out that co-carrier-based SC is more reliable compared to the co-carrier-based DC.

### 4.3. Impact of Selection Time-Slot in Co-Carrier-Based SC

In downlink time division multiplexing (TDM) SATCOM, the system would allocate transmission time-slots for the selection process in the co-carrier-based SC method. The length of each time-slot could be adjusted according to the transmission rate. Generally speaking, lower carrier rates require a longer time-slot. In this subsection, we compare the advancement of DC and SC methods in terms of the length of time-slot τ.

**Definition 1.**  *For the beam-edge UT, the ergodic rate gain of the co-carrier-based SC method is defined as*

(39) $$\begin{array}{c} G=10\log\frac{E[R_{k}^{{\;}^{\prime }}]}{E[R_{k}]}. \end{array}$$

To obtain a positive value of *G*, $E\left[R_{k}^{{\;}^{\prime }}\right]-E\left[R_{k}\right]>0$ should be met. In this case, the length of each time-slot satisfies

(40) $$\begin{array}{c} \tau <\frac{\int_{0}^{\infty }\frac{1-{\left(1-e^{-\frac{x}{|\beta_{k}{|}^{2}\delta \left(1-\alpha x\right)}}\right)}^{M}}{1+x}dx}{M}-\delta |\beta_{k}{|}^{2-2M}\sum_{i=1}^{n}\omega_{i}B_{k}\left(\delta M\right|\beta_{k}{|}^{2}x_{i}). \end{array}$$

## 5. Simulation Results

In this section, we run simulations in MATLAB and provide simulation results in terms of the ergodic rate and outage probability. Since the LEO SATCOM would be characterized as multiple coverage in the future, we set M=6 and K=3 throughout the simulation. That is, each beam-edge UT could be covered by six satellites. The normal gain of transmit beams onboard is assumed as $E_{S}=45$ dB. Let $\eta_{3dB}=2.5^{\circ }$ and $\eta_{m,m}=0.1^{\circ }$ for the beam-center UT. Then, $\eta_{k,m}=\eta_{3dB}=2.5^{\circ }$ for the beam-edge UT. We assume the system works in L band where f=1 GHz. The orbit height is d=700 km. In this case, $d_{m,m}=700$ km and $d_{k,m}=d/\cos\left(\eta_{k,m}\right)$.

For comparison reasons, we provide simulation results of the following schemes:

- “ESR-DC, sim”: numerical simulation of ergodic sum-rate for co-carrier-based DC;
- “ESR-SC, sim”: numerical simulation of ergodic sum-rate for co-carrier-based SC;
- “ESR-non, sim”: numerical simulation of ergodic sum-rate without cooperative transmission;
- “ER-DC-edge, sim”: numerical simulation of ergodic rate for UT *k* in co-carrier-based DC;
- “ER-SC-edge, sim”: numerical simulation of ergodic rate for UT *k* in co-carrier-based SC;
- “ER-non-edge, sim”: numerical simulation of ergodic rate for UT *k* without cooperative transmission;
- “ESR-DC, exp”: analytical expression of ergodic sum-rate for co-carrier-based DC;
- “ESR-SC, exp”: analytical expression of ergodic sum-rate for co-carrier-based SC;
- “ER-DC-edge, exp”: analytical expression of ergodic rate for UT *k* in co-carrier-based DC;
- “ER-SC-edge, exp”: analytical expression of ergodic rate for UT *k* in co-carrier-based SC.
- “OP-DC-edge, sim”: numerical simulation of outage probability for UT *k* in co-carrier-based DC;
- “OP-SC-edge, sim”: numerical simulation of outage probability for UT *k* in co-carrier-based SC;
- “OP-non-edge, sim”: numerical simulation of outage probability for UT *k* without cooperative transmission;
- “OP-center, sim”: numerical simulation of outage probability for beam-center UT;
- “OP-DC-edge, exp”: analytical expression of outage probability for UT *k* in co-carrier-based DC;
- “OP-SC-edge, exp”: analytical expression of outage probability for UT *k* in co-carrier-based SC;
- “OP-center, exp”: analytical expression of outage probability for beam-center UT.

Specifically, “ESR-non”, “ER-non-edge”, and “OP-non-edge” are considered as benchmarks where the beam-edge UT randomly accesses one of the satellites without using the cooperative transmission [14,15]. In addition, the co-carrier scheme is still applied in the above baseline methods.

### 5.1. Ergodic Rate

**Figure 4:**
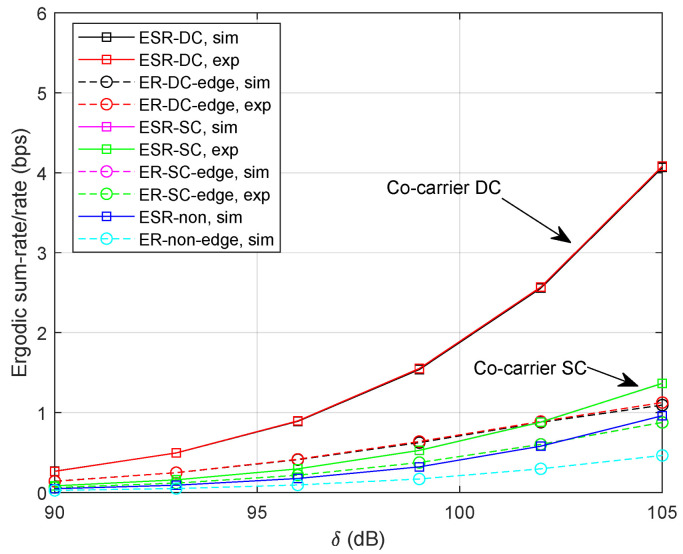
Ergodic sum-rate/rate with τ=1 ms and α=0.4.

Figure 4 illustrates the simulation results with δ=[90,105] dB. Specifically, the length of selection time-slot is set as τ=1 ms and the power factor is α=0.4. It can be clearly noticed that the curves of analytical expressions closely follow with the numerical simulations. Since signals for a beam-edge UT and *M* beam-center UTs can be transmitted simultaneously in the co-carrier-based DC method, we can expect a more promising ergodic sum-rate compared with the co-carrier-based SC method. For the beam-edge UT, we can find out that the co-carrier-based DC scheme outperforms the co-carrier-based SC method in the low SNR region. However, the result of co-carrier-based SC method approaches that of DC when the channel becomes better. Moreover, the ergodic sum-rate or the rate for beam-edge UT could be improved by using either method compared with a non-cooperative strategy.

**Figure 5:**
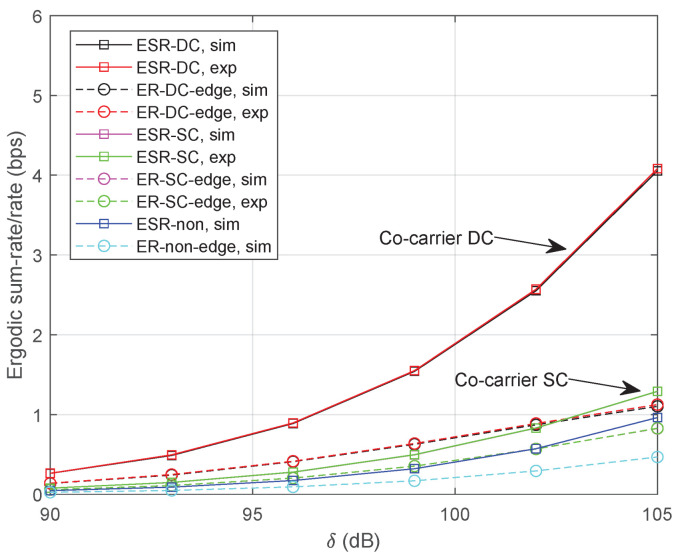
Ergodic sum-rate/rate with τ=10 ms and α=0.4.

To see how the co-carrier-based SC method behaves with a selected time-slot, we provide the results with τ=10 ms. It is noteworthy that the advantage of co-carrier-based SC fades away with the growing time-slot shown in Figure 5. That is, the higher transmission rate is beneficial to the co-carrier-based SC method.

**Figure 6:**
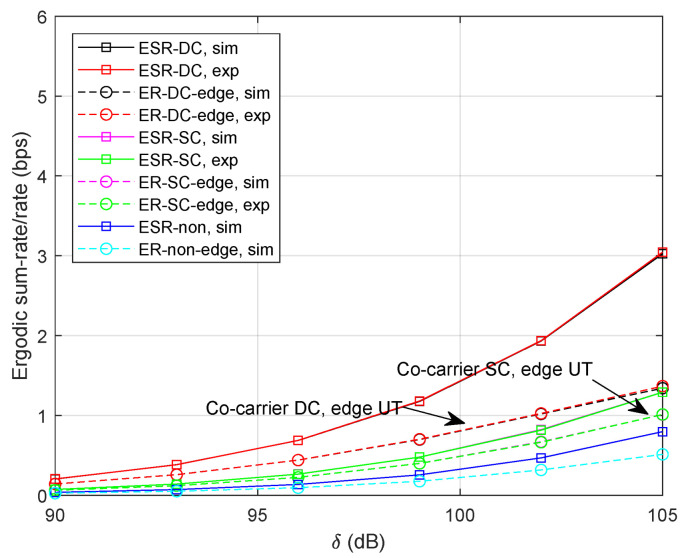
Ergodic sum-rate/rate with τ=1 ms and α=0.2.

Figure 6 illustrates the ergodic rates with α=0.2. That is, the signal power for beam-center UTs lowers while the interference for beam-edge UTs reduces as well. It is observed that the performance of the co-carrier-based DC degrades significantly since there exist *M* beam-center UTs in each transmission slot. We can clearly find out that the ergodic rate for the beam-edge UT in both methods increases compared with the result in Figure 4. Additionally, the ergodic sum-rates of co-carrier-based SC and non-cooperative transmission also degrade under the influence of lower power factors.

**Figure 7:**
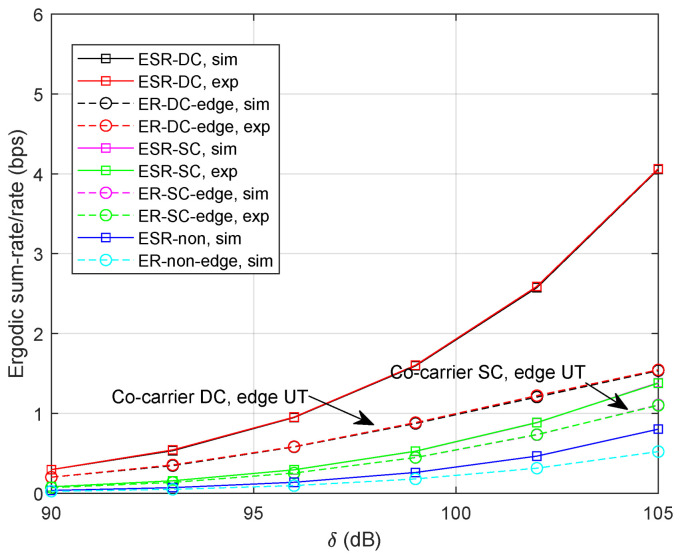
Ergodic sum-rate/rate with M=9, τ=1 ms and α=0.2.

Figure 7 shows the results of ergodic rate with more visible satellites where M=9. It can be noticed that the ergodic sum-rate of the co-carrier-based DC could be significantly improved since more simultaneously served beam-center UTs are involved. In addition, the ergodic rate of the beam-edge UT also increases since more signal paths are received at the UT. Note that there is no noticeable performance improvement for the co-carrier-based SC method.

### 5.2. Outage Probability

**Figure 8:**
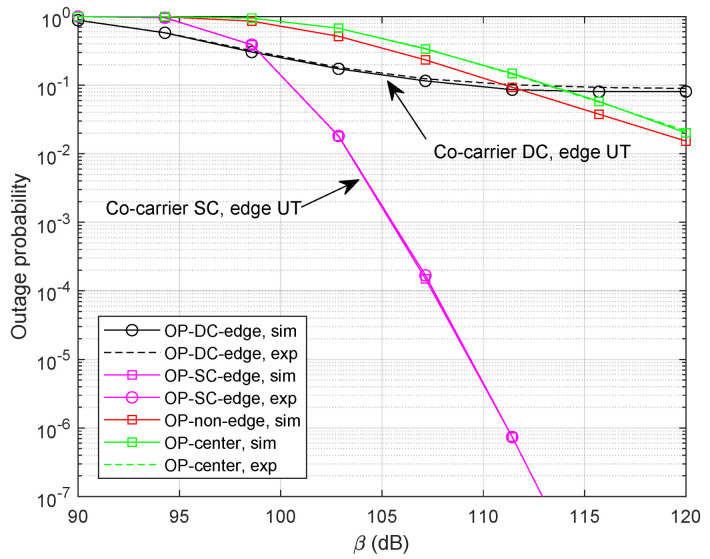
Outage probability with $R_{m}=0.4$ bps, $R_{k}=0.3$ bps, and α=0.4.

Let $R_{m}=\log\left(1+\Gamma_{m}\right)$ and $R_{k}=\log\left(1+\Gamma_{k}\right)$ denote the achievable rate of beam-center UT and beam-edge UT at thresholds $\Gamma_{m}$ and $\Gamma_{k}$, respectively. We provide simulation results with $R_{m}=0.4$ bps and $R_{k}=0.3$ bps, shown in Figure 8. As expected, the curves of analytical expressions closely follow with those of numerical simulations. It can be clearly observed that the co-carrier-based SC can provide a promising outage probability performance compared with the co-carrier-based DC method since the SC strategy is able to choose the best signal path. Specifically, we can find out that the diversity order of the co-carrier-based DC is zero which illustrates the DC method could not offer additional gain in high SNR regions.

**Figure 9:**
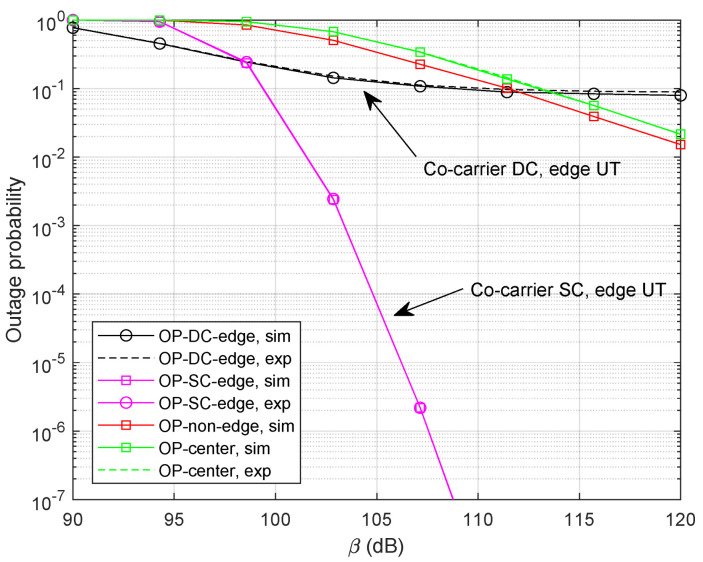
Outage probability with M=9 and α=0.4.

Next, we set M=9 and study the benefit of *M* for both methods. It can be clearly noticed in Figure 9 that the outage performance for the co-carrier-based SC method can be significantly improved with more optional LEO satellites. Unfortunately, the increase in *M* could not provide extra diversity gain for the co-carrier-based DC scheme. Since no selection is involved in the demodulation of beam-center UTs, the outage performance could not be relieved either.

**Figure 10:**
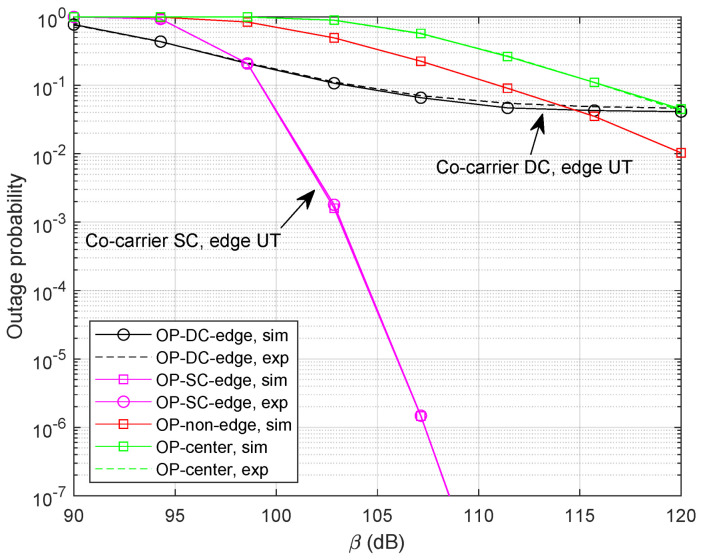
Outage probability with M=9 and α=0.2.

Figure 10 shows the outage probability with a lower power factor, α=0.2. It is observed that the performance of the beam-edge UT has been improved in both methods. As a consequence, the results of the beam-center UT degrade. Again, the superiority of the SC scheme is obvious and the DC method works well in low SNR regions.

## 6. Conclusions

Taking full advantage of the multiple coverage characteristic of LEO constellation, this paper proposed two cooperative downlink transmission methods. In detail, beam-edge UTs could directly combine the signals as one useful signal from the accessible satellites in the DC method. With the help of DC, signals arrived at the UT could be enhanced. When the system applies TDM in downlink transmission, beam-edge UTs are able to receive signals in different time-slots from the satellites. To obtain a better performance, we further study an SC-based method where the receiver at the UT would select the strongest signal to demodulate. However, the selection overhead is not friendly toward the ergodic sum-rate. Specifically, as there exists a significant equivalent channel gain gap at the beam-edge and beam-center, we exploited the co-carrier scheme for both methods where the beam-edge and beam-center UTs are able to occupy the same resource block. To analyze the ergodic sum-rate and outage probability, we further derive the analytical expressions for the new methods. Simulation results illustrate that the co-carrier-based DC could provide a remarkable ergodic sum-rate or ergodic rate for beam-edge UTs. In terms of the outage probability, the co-carrier-based SC method outperforms the DC since the diversity order of the SC increases with the number of the satellites. Overall, the proposed co-carrier cooperative methods could fully use the energy resource without consuming additional power onboard compared with the non-cooperative method.

## Figures and Tables

**Table 1:** Symbols of the system.

The number of the visible satellites in the overlapping area	*M*
The number of the UTs in the overlapping area	*K*
The number of array elements along the *y* axis	$N_{y}^{S}$
The number of array elements along the *z* axis	$N_{z}^{S}$
The radius of the overlapping area	*r*

## Data Availability

The data presented in this study are available on request from the corresponding author.
